# Unravelling the evolutionary dynamics of antibiotic resistance genes in the infant gut microbiota during the first four months of life

**DOI:** 10.1186/s12941-024-00725-z

**Published:** 2024-08-13

**Authors:** Anna Samarra, Raúl Cabrera-Rubio, Cecilia Martínez-Costa, Maria Carmen Collado

**Affiliations:** 1 Departament of Biotechnology, Institute of Agrochemistry and Food Technology- National Spanish Research Council (IATA-CSIC), Paterna, Valencia Spain; 2 Department of Pediatrics, School of Medicine, University of Valencia, Valencia, Spain; 3 Pediatric Gastroenterology and Nutrition Section, Hospital Clínico Universitario Valencia, INCLIVA, Valencia, Spain

**Keywords:** Antibiotic resistance, Infant, Gut, Breastfeeding, Microbiota

## Abstract

**Background:**

Alongside microbiota development, the evolution of the resistome is crucial in understanding the early-life acquisition and persistence of Antibiotic Resistance Genes (ARGs). Therefore, the aim of this study is to provide a comprehensive view of the evolution and dynamics of the neonatal resistome from 7 days to 4 months of age using a high-throughput qPCR platform.

**Methods:**

In the initial phase, a massive screening of 384 ARGs using a high-throughput qPCR in pooled healthy mother-infant pairs feces from the MAMI cohort was carried out to identify the most abundant and prevalent ARGs in infants and in mothers. This pre-analysis allowed for later targeted profiling in a large number of infants in a longitudinal manner during the first 4 months of life. 16S rRNA V3-V4 amplicon sequencing was performed to asses microbial composition longitudinally. Potential factors influencing the microbiota and ARGs in this period were also considered, such as mode of birth and breastfeeding type.

**Results:**

Following the massive screening, the top 45 abundant ARGs and mobile genetic elements were identified and studied in 72 infants during their first months of life (7 days, 1, 2, and 4 months). These genes were associated with resistance to aminoglycosides, beta-lactams and tetracyclines, among others, as well as integrons, and other mobile genetic elements. Changes in both ARG composition and quantity were observed during the first 4 months of life: most ARGs abundance increased over time, but mobile genetic elements decreased significantly. Further exploration of modulating factors highlighted the effect on ARG composition of specific microbial genus, and the impact of mode of birth at 7 days and 4 months. The influence of infant formula feeding was observed at 4-month-old infants, who exhibited a distinctive resistome composition.

**Conclusions:**

This study illustrates the ARG evolution and dynamics in the infant gut by use of a targeted, high-throughput, quantitative PCR-based method. An increase in antibiotic resistance over the first months of life were observed with a fundamental role of delivery mode in shaping resistance profiles. Further, we highlighted the influence of feeding methods on the resistome development. These findings offer pivotal insights into dynamics of and factors influencing early-life resistome, with potential avenues for intervention strategies.

**Supplementary Information:**

The online version contains supplementary material available at 10.1186/s12941-024-00725-z.

## Background

Antibiotic resistance, a complex and pressing global health crisis, continues to challenge our ability to combat infectious diseases effectively. The emergence and transfer of antibiotic resistance genes within bacterial populations have brought attention to the concept of the "resistome," a vast reservoir of genetic elements responsible for conferring resistance to antibiotics [1]. Antibiotic resistance continues to challenge our ability to combat infectious diseases as once-effective antimicrobial agents become increasingly ineffective, leading to longer hospital stays, higher mortality rates, and escalating healthcare costs [2]. This becomes particularly dangerous in early stages of life, where the acquisition of antibiotic resistances has short and long term consequences on the infant health.

Infancy is a critical period characterized by rapid microbial acquisition and colonization of the gut with potential impacts on health outcomes [3]. During early life, the gut microbiota is highly dynamic and variable, influenced by a multitude of intrinsic and extrinsic factors [4–6]. Among these factors, antibiotic exposure, either through maternal transmission or direct administration, is recognized as a significant driver of antibiotic resistance gene dissemination and selection within the infant gut microbiome [7, 8]. Other factors, such as breastfeeding practices and mode of delivery have also been associated with the establishment of the infant gut resistome [9–11]. Consequently, deciphering the dynamics of the infant gut resistome during the lactation period has become a matter of importance, as it sets the stage for continued microbial colonization and immune development in early life.

Despite variations in the abundance and diversity of antibiotic resistance among different infant groups due to varying early-life factors, prevailing resistance genes primarily encode for proteins that provide resistance against beta-lactams, tetracyclines, macrolides, aminoglycosides, and quinolones [12]. Infant resistome analyses at different time points have elucidated greater relative abundances of antibiotic resistance genes (ARGs) in younger infants compared to older children [13–16]. Thus, the development of the infant resistome is linked to the evolution of the microbial composition, contributing to the long-term dynamics and stabilization of the resistome into adulthood [17].

The potential risks posed by antibiotic resistant bacteria in samples can be estimated quantitatively with high-throughput qPCR. This technique is widely used to determine the presence and abundance of multiple ARGs in environmental microbiota and in various types of samples [18–22]. High-throughput qPCR can be easily customized to target specific ARGs of interest, allowing us to focus on clinically relevant or ecologically significant resistance genes [23].

Hence, quantitatively studying the evolution of the infant gut resistome is crucial for understanding the early-life acquisition and persistence of antibiotic resistances and the role of lactation and other factors on these resistances. For this reason, the aim of this research is to provide a comprehensive overview of the evolution and dynamics of the infant resistome, from 7 days to 4 months of age, using a high-throughput qPCR platform. By deepening our knowledge of the infant gut resistome, we can pave the way for targeted interventions aimed at mitigating the spread of antibiotic resistance during this critical developmental period.

## Materials and methods

### Study design and cohort

**Fig. 1:**
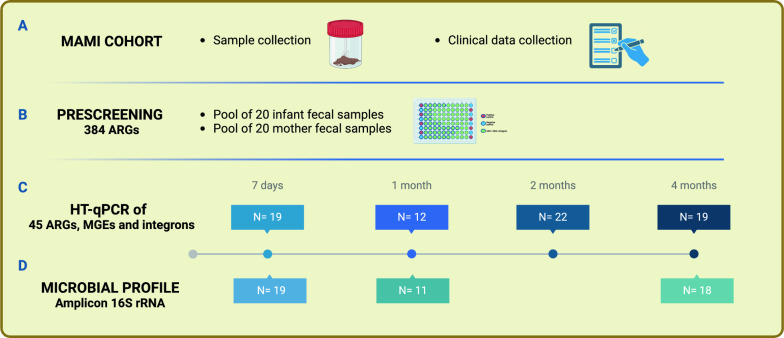
Flowchart of the study design. A total of 32 mother-infant pairs were included for this study, from which clinical data and fecal samples were collected. In the initial phase, a massive screening of 384 ARGs in pooled infant and maternal feces from the MAMI cohort was carried out, separately, to identify the most abundant and prevalent ARGs. This pre-analysis allowed later targeted profiling in a large number of infants in a longitudinal manner during the first 4 months of life. 16S rRNA V3-V4 amplicon sequencing was performed to assess microbial composition longitudinally. Exact number of samples for each analysis (N) are shown in the illustration

A total of 32 mother-infant pairs from the MAMI birth cohort [24] participated in this study, taking a total of longitudinal 72 infant fecal samples collected at 7 days, 1 month, 2 months and 4 months of age (Fig. 1). Maternal-infant clinical data including gender, mode of delivery, breastfeeding practices (exclusive breastfeeding or formula feeding, which included both mixed and formula-feeding) and duration, antibiotic exposure, gestational age, and anthropometric measures were collected (Table 1).

**Table 1:** Characteristics of study participants

Mother characteristics	Total (*N* = 32)
Gestational age (weeks)	39.5 [39, 40]
Weight gain over the pregnancy (kg)	11.46 ± 3.891
Pre-gestational BMI (kg m − 2)	23.7 ± 3.6
Antibiotic consumption during pregnancy (%)	9 (28.12%)
Mother charateristics	
Gender: female (%)	32 (44.4%)
Birth mode: vaginal birth (%)	47 (65.3%)
Duration of breastfeeding (months)	6 [3–12]
Exclusive breastfeeding (%)	
7 days	27 (84.4%)
1 month	19 (59.4%)
2 months	18 (56.3%)
4 months	17 (53.1%)
Antibiotic exposure (%)	
At birth	13 (40.6%)
7 days	3 (9.4%)
1 month	2 (6.3%)
2 months	1 (3.1%)
4 months	1 (3.1%)
BMIZ	
At birth	(−) 0.27 ± 1.1
1 month	(−) 0.50 ± 0.9
6 months	1.10 ± 0.3

Categorical variables are expressed as positive cases-prevalence and (percentage, %). Normally distributed data are presented as mean ± standard deviation (SD) and non-normal data as median and interquartile range [IQR]. BMIZ, body mass index z-score. Infant body length and weight was only recorded at moment of birth, 1 month and 6 months of age. N = 19 of 7 days old infants, N = 12 of 1 month old infants, N = 22 of 2 months old infants and N = 19 of 4 months old infants

### Maternal-infant biological samples and DNA extraction and quantification

The neonatal samples were collected as described previously [5] in sterile containers by the parents at home using detailed instructions (Fig. 1A). Stools were collected at 7 days, and 1, 2 and 4 months after birth and immediately stored at − 20 °C. Maternal fecal samples were self-collected at 2 months postpartum following specific instructions as described previoulsy [24]. Within 24 h of collection, samples were taken to Primary Health Care Centers (during scheduled pediatric visits). There they were stored at − 80 °C until analysis.

Total DNA was extracted, purified, and quantified as described in Selma-Royo et al [5]. Briefly, DNA was extracted from 50 to 100 mg of fecal material using the Master-Pure DNA extraction Kit (Epicentre, Madison, WI, USA) following the manufacturer’s instructions with the following modifications: treatment with lysozyme (20 mg/mL) and mutanolysin (5 U/mL) for 60 min at 37 °C and a preliminary step of cell disruption with 3-μm diameter glass beads for 1 min at 6 m/s by a FastPrep 24-5G Homogenizer bead beater (MP Biomedicals). Purification of the DNA was performed using a DNA Purification Kit (Macherey–Nagel, Duren, Germany) according to the manufacturer’s instructions. DNA concentration was measured using Qubit® 2.0 Fluorometer (Life Technology, Carlsbad, CA, USA) for further analysis.

### Screening of the top antibiotic resistance genes in the maternal-infant gut microbiota

A pool DNA from of 20 infant fecal samples (1 ng) taken at 2 months of age and a pool of DNA from their respective mothers (1 ng) at the same timepoint were used to pre-screen for the positive detection of 384 previously validated ARGs using the SmartChip Real-Time PCR (Resistomap, Finland) (Fig. 1B and Supplementary Table 1) [25]. The 16S rRNA gene was used as a positive control and to normalize the abundances of detected genes in fecal samples. Within the chip, each primer set was designed to target sequence diversity within a gene to assess the gene's variants in the resistome. Therefore, each gene was analysed independently. Briefly, SmartChip has 5184 reaction wells with a volume of 100 nL. Each 100 nL reaction is comprised of 1 × SmartChip TB Green Gene Expression Master Mix (TakaraBio), nuclease-free PCR-grade water, 300 nM of each primer and a DNA template of 2 ng/μL. The chips are filled using the SmartChip Multisample Nanodispenser (Takara Bio). qPCR cycling conditions were maintained and initial data processing was done as previously described [26]. qPCR reactions were performed with three technical replicates with a limit of detection of cycle threshold (CT) = 27 [26–28]. A gene was considered present in a sample if it was detected in at least two of the three replicates. The mean CT values of the replicates for each reaction was used to calculate the ΔCT values (ΔCT = CT detected gene – CT 16S rRNA gene), and the 2 − ΔCT method was used to calculate the relative abundances of the detected gene relative to the 16S rRNA gene in each sample [28]. The 45 most abundant and representative ARGs, MGEs and integrons present in the infant gut of our cohort were intentionally chosen for targeted quantification for this study to guarantee their consistent detection across all samples.

### Gut microbiota profiling by targeted 16S rRNA amplicon sequencing

We also carried out the microbiota profiling of a subset of 48 infant fecal samples by amplification of the V3-V4 regions of the 16S rRNA gene (Fig. 1D). Amplicons were obtained with PCR amplification using barcoded conventional primers (341F 5′-CCTACGGGNGGCWGCAG-3′ and 806R 5′GGACTACNNGGGTATCTAAT-3′) with a 466 bp fragment length. Amplicons were checked with a Bioanalyzer DNA 1000 chip and libraries were sequenced using a paired-end kit on a MiSeq-Illumina platform (FISABIO sequencing service, Valencia, Spain). The 63754 (± 33733.52) mean sequences with good quality were obtained from samples. Overall, these sequences were clustered into 87 ZOTUs (± 14). Bacterial diversity analysis was done using raw reads, which were quality controlled and filtered (Quality: 25 and length: 150 bp) using trimGalore (v0.6.4_dev; https://github.com/FelixKrueger/ TrimGalore). The paired-end reads with a minimum overlap of 30 bp were joined using Fastq-join [29]. Sequences were trimmed of primers and distal bases, and singletons were removed with USEARCH v11 [30]. zOTUs (zero-radius operational taxon units) mapping to the human genome (GRCh38) using the Burrow–Wheeler Aligner in Deconseq v0.4.3 were filtered out. The resulting reads were denoised and chimeras were filtered with UNOISE3 [31]. Taxonomic assignment of zOTUs was performed in QIIME2 v2018.2 [32] using the QIIME2 feature classifier plugin [33] and the Ribosomal Database Project (RDP 2.12) [34]. The zOTUs were aligned with MAFFT [35] to then make a phylogenetic tree with FASTTREE [36] that was midpoint-rooted.

### Statistical analysis

All statistical analyses were performed with R version 4.1, and figures were drawn with the “ggplot2” R package [37]. Variables are presented as the number (percentage) or mean (standard deviation, SD), as appropriate. Statistical analyses for comparison of clinical and perinatal characteristics included the use of non-parametric Mann–Whitney U tests or Fishers exact test and Pearson χ^2^ tests for continuous and categorical variables, respectively, to compare different groups of infants. Normality of the data was evaluated with Shapiro–Wilk tests.

Antibiotic resistance genes statistical tendency through the multiple timepoints was calculated with Wilcoxon test. The Shannon index for alpha diversity analyses of the relative abundances of the genes was calculated using the *diversity* function of the “*vegan*” R package [38]. Principal Component Analysis (PCA) was conducted to study the composition of ARGs between all timepoints and to confirm the correlation between environmental factors and ARG composition, using the ‘factoextra’ [39] and ‘FactoMineR’ [40] R packages. We tried to identify groups of variables whose balance was more associated with the response variable, using the R package MaAsLin2 [41] which is a complete R package to efficiently determine multivariable associations through general linear models that can accommodate most modern study designs.

Calculations of microbial richness (Observed, Chao1 and ACE) and evenness indices (Shannon and Simpson) were done using the “phyloseq” R package [42]. Beta diversity was characterised by Principal Coordinate Analysis (PCoA) conducted by plotting the Bray–Curtis distance matrix of log transformed zOTU counts for each timepoint separately. We filtered the zOTU data using the *filter_taxa* function in the “phyloseq” package, and only zOTUs present in at least 10% of samples were retained. The *adonis* permutational test was used to evaluate overall differences in microbiota structure between the timepoints with the “*vegan*” R package [38]. We also fitted a Poisson (log-linear regression) generalized lineal mixed model (GLMM) using the “*mvabund*” package [43] to assess the association of the top 10 most abundant taxa individually adjusted by time, mode of delivery and mode of lactation, on the antibiotic resistance gene composition. For all methods, p-values were adjusted for multiple comparisons using False Discovery Rate (FDR) based on Benjamini–Hochberg (BH) [44].

## Results

### Pre-screening for top antibiotic resistances in the maternal-infant gut microbiota 

In this study, we identified a set of ARGs with the highest values in the maternal-infant gut from a pre-screening analysis. A total of 211 ARGs were identified in the infants guts, while only 135 were detected in the mothers guts. The overall antibiotic resistance abundance was higher in 2-month-old infants than in their mothers, and statistically significant in the case of mobile genetic elements (MGE, 0.008 ± 0.032 in infant vs 0.0005 ± 0.003 in mothers, p < 0.0001) and aminoglycosides (0.0018 ± 0.007 in infants vs 0.0006 ± 0.001, p < 0.0001; Table 2).

**Table 2:** Results of the antibiotic resistance genes detected in the pre-screening of the mother and infant fecal sample pools

		Genes detected (%)	Relative abundance^#^	p-value
Integrons (N = 4)	Infant	100.00	0.043 [0.021–0.121]	0.859
	Mother	75.00	0.004 [0.004–0.042]	
MGE (N = 48)	Infant	60.41	0.008 [0.001–0.038]	< 0.0001*
	Mother	43.75	0.0005 [0.0002–0.003]	
Betalactams (N = 54)	Infant	46.29	0.0006 [0.0002–0.003]	0.397
	Mother	18.51	0.0004 [0.0002–0.001]	
MLSB (N = 47)	Infant	53.19	0.001 [0.0005–0.007]	0.871
	Mother	29.78	0.003 [0.0002–0.009]	
Aminoglycoside (N = 60)	Infant	58.33	0.001 [0.0003–0.007]	0.035*
	Mother	41.66	0.0006 [0.0002–0.001]	
MDR (N = 39)	Infant	64.10	0.001 [0.0008–0.0204]	0.201
	Mother	35.89	0.002 [0.0001–0.003]	
Sulfonamide (N = 6)	Infant	100.00	0.004 [0.0007–0.01]	0.322
	Mother	66.66	0.0004 [0.0001–0.001]	
Phenicol (N = 22)	Infant	45.45	0.0004 [0.0002–0.002]	0.943
	Mother	18.18	0.0005 [0.0001–0.001]	
Tetracycline (N = 26)	Infant	69.23	0.008 [0.002–0.0171]	0.216
	Mother	53.84	0.0009[0.0002–0.0131]	
Quinolone (N = 11)	Infant	54.54	0.001 [0.0005–0.003]	0.401
	Mother	18.18	0.0006 [0.0003–0.0008]	
Other (N = 17)	Infant	52.94	0.0017[0.0002–0.0119]	0.710
	Mother	41.17	0.005 [0.002–0.008]	
Vancomycin (N = 24)	Infant	33.33	0.001 [0.0008–0.002]	0.008*
	Mother	20.83	0.0001 [0.0001–0.0001]	
Trimethoprim (N = 17)	Infant	47.05	0.0002 [0.0001–0.0003]	–
	Mother	0.00	–	

^#^Relative abundances of the detected genes relative to the 16S rRNA gene in each sample. Normally distributed data are presented as mean ± standard deviation (SD) and non-normal data as median and interquartile range [IQR]. Pearson’s-Chi-square test was used for categorical variables, and Mann–Whitnney U test (or Fisher’s exact test) were used for continuous variables, as appropriate, for calculating statistical significance, which are marked with a *p < 0.05 was considered statistically significant

The 45 most abundant ARGs, MGEs and integrons present in the infant gut were intentionally chosen for this study to guarantee their consistent detection across all samples (Table 3). The targeted genes we included in the customized SmartChip were the total 16S rRNA gene, specific genes for the Bacteroides and Firmicutes phyla, and the 45 antibiotic ARGs, MGEs, integrons, and other genes associated with antibacterial compounds. Notably, genes from different functional groups appeared in this set of 45 genes, highlighting the diverse genetic elements and mechanisms at play in the early infant gut (Supplementary Table 2).

**Table 3:** List of antibiotic resistance genes (ARGs) measured in the infant fecal samples

Antibiotic class	ARG
Integrons (N = 1)	*intI1_1*
MGE (N = 3)	*IS26_1*, *tnpA_1*, *ISEcp1*
MLSB (N = 6)	*ermX_2*, *mphA*, *ermF*, *oleC* *mefA*, *ermB_3*
Quinolone (N = 3)	*qepA,* *qnrS_1*, *qnrB*
Aminoglycosides (N = 6)	*aph4-ib*, *strB,* *aac(6')-Ib_1*, *aacC2*, *apmA*, *aadA7*
MDR (N = 4)	*mdtH*, *czcA*, *pcoA*, *tolC_2*
Betalactams (N = 10)	*blaCTX-M*, *blaTEM*, *blaOXY*, *penA*, *blaACT*, *blaSHV11,* *blaSFO*, *pbp*, *cfxA,* *blaOXA48*
Other (N = 2)	*bacA*, *mcr1*
Phenicol (N = 3)	*mdtL*, *cmlV*, *catA1*
Vancomycin (N = 3)	*vanA*, *vanHB*, *vanB_1*
Tetracycline (N = 4)	*tetO_2*, *tetA_2*, *tetW*

### Early-life antibiotic resistance composition changes over time

**Fig. 2:**
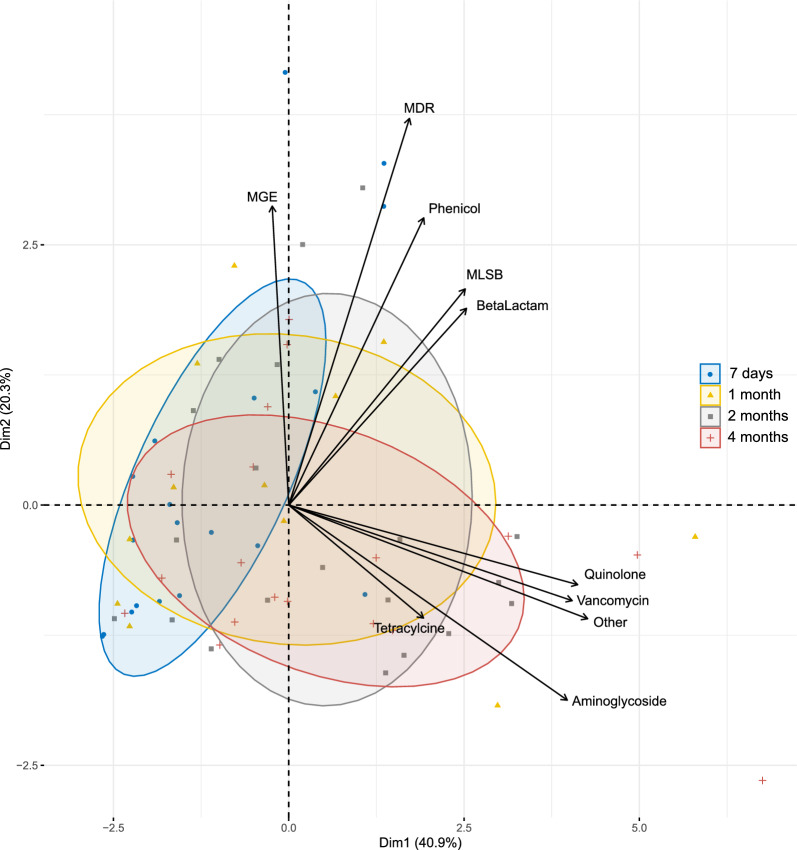
Antibiotic resistance composition changes over time. Principal Coordinate Analysis (PCA) biplot depicting the relationship between the composition of antibiotic resistances and time. Blue ellipses represent 7-days old infants, yellow ellipses the 1-month old, grey ellipses the 2-months old, and red ellipses the 4-months old infants. The black arrows are the group of ARGs that best explain the differences between the sample groups. Angles between the arrows represent correlations; acute angles represent positive correlations and obtuse angles represent negative correlations

The diversity of antibiotic resistance genes was found to increase significantly though time, going from a Shannon index of 1.81 ± 0.842 in 7-day-old infants, to 1.969 ± 0.612 at 1 month of age, 2.468 ± 0.495 at 2 months, and 2.397 ± 0.312 at 4 months (p < 0.05 between all timepoints). The antibiotic resistance gene composition was also compared between all timepoints (Fig. 2) and differed significantly between 7 days and 4 months of age (p = 0.024). Indeed, MGEs were highly correlated to 7-day-old samples (p < 0.001), whereas ARGs conferring resistance to quinolones, vancomycin and aminoglycosides were significantly correlated to 4-month-old samples (p < 0.001).

**Fig. 3:**
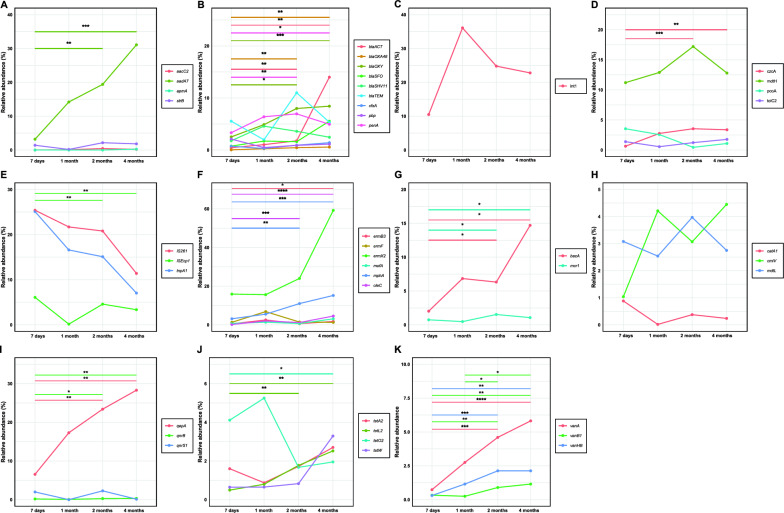
Evolution of antibiotic resistance genes relative abundance. Tendency plots representing the antibiotic resistant genes evolution from 7 days to 4 months of age of each ARGs measured, grouped by antibiotic resistance to: **A** Aminoglycosides, **B** Beta-lactams, **C** Integrons, **D** MDR, **E** MGE, **F** MLSB, **G** Others, **H** Phenicol, **I** Quinolone, **J** Tetracycline and **K** Vancomycin. Statistical differences are marked as following: *p < 0.05, **p < 0.01, ***p < 0.001, ****p < 0.0001

The evolution of each individual ARG over time was reported (Fig. 3 and Supplementary Fig. 1) and detailed results are described below.

#### Aminoglycosides

Aminoglycoside evolution was tested by quantifying the relative abundances of the *aaC2, aadA7, apmA* and *strB* genes (Fig. 3A). The relative abundance of the aminoglycoside resistant gene *aadA7* shows a significant increase from 7 days to 2 months of age (3.20–19.44%; p < 0.01) and 4 months (31.11%; p < 0.001). At 2 and 4 months of age, *aadA7* has a significantly higher relative abundance than the other aminoglycoside resistance genes (p < 0.05). Moreover, at 2 months, the *strB* is more abundant than *aacC2* gene (2.15% and 0.43%; p = 0.004).

#### Beta-lactams

A total of 9 ARGs conferring resistance to antibiotics where measured (Fig. 3B). A significant increase from 7 days to 2 months and to 4 months of age was observed for *blaOXA48* (0.04–0.45%; p < 0.01, and to 0.56%; p < 0.01), *blaACT* (044–1.77%; p < 0.01, and to 14.04%; p < 0.01) and *blaOXY* (2.52–4.98%; p < 0.05, and to 7.41%; p < 0.001)*.* The *penA* resistance gene showed a significant increase from 7 days to 2 months (3.33–6.94%; p < 0.05) but then decreases at 4 months (4.94%; p < 0.01), althouth it keeps statistically higher compared to 7 days abundance.

Both 2- and 4-month-old infants have higher amounts of the *blaOXY* gene (7.98% and 8.41%, respectively) than *blaOXA48* (0.45%; p = 0.003, and 0.56%; p = 0.002)*, cfxA* (0.92%; p = 0.001 and 1.41%; p = 0.016) and *pbp* (0.89%; p = 0.012 and 1.14%; p = 0.024), whereas *penA* is in higher amounts than *blaOXA48* (p = 0.006 and p = 0.015) and *cfxA* (p = 0.003 and p = 0.032)*.* The resistant gene *cfxA* has a lower abundance in 2-month-old infant guts than *blaSHV11* (3.59%; p = 0.007), *blaSFO* (1.60%; p = 0.015), and *blaACT* (1.77%; p = 0.013). Finally, 2-month-old infants had higher abundances of *penA* than *pbp* (p = 0.015), and *blaSHV11* than *blaOXA48* (p = 0.04).

#### Integrons

Integron abundance in infants’ guts was measured by quantifying the *int1* integron, which showed an increase from 7 days (10.47%) to 1 month of age (36.12%), and then a decrease at 2 (24.84%) and 4 months of age (22.84%), but no statistical differences were found between time points (Fig. 3C).

#### Multi drug resistances (MDR)

*czcA, mdtH, pcoA* and *tolC2* were measured to assess the evolution of multi-drug resistant genes. *mdtH* increased significantly from 7 days (11.20%) to 2 months of age (17.22%; p < 0.001), but then decreased when infants reached 4 months of age (12.77%; p < 0.01). This gene is highly more abundant than *tolC2* and *pcoA* genes in 2- and 4-month-old infants (1.23%; p = 0.06 and 1.77%; p = 0.004 for 2 months, and 0.44%; p < 0.0004 and 1.09%; p = 0.002, for 4 months, respectively for each gene). Finally, 2-month-old infants had lower amounts of *pcoA* than *czcA* (3.53%; p = 0.015) in their gut (Fig. 3D).

#### MGE

Transposase *tnpA1* and insertion sequences *IS261* and *ISEcp1* were measured as representative for the MGE group (Fig. 3E). All three of these genes showed a decrease from 7 days to 4 months of age, with *ISEcp1* significant between 7 days and 2 months (6.09% and 4.58%; p < 0.01) and between 7 days and 4 months (3.36%; p < 0.01).

#### Macrolide-lincosamide-streptogramin B (MLSB)

The six macrolide-lincosamide-streptogramin antibiotic resistance genes measured were: *ermB3, ermF, ermX2, mefA, mphA* and *oleC* (Fig. 3F). Similar to other groups of antibiotics, we observed an increase in the abundance of *mphA* and *oleC* genes from 7 days to 2 months of age (3.14–11.02%; p < 0.01 and 0.18–1.20%; p < 0.001, respectively). The same pattern was observed from 7 days to 4 months of age (3.14–11.02%; p < 0.001 for *mphA,* 0.18–4.52%; p < 0.01 for *oleC,* and 3.14–11.02%; p < 0.001 for *ermB3*). This increase longer-range increase was also observed for the *ermB3* gene (from 0.53% at 7 days to 1.76% at 4 months of age; p < 0.05). *ermX2* was significantly more abundant at 2 and 4 months of age (23.97% and 59.18%, respectively) than *oleC* (p = 0.0005 and p = 0.009, respectively for each timepoint), *mefA* (p = 0.0002 and p = 0.006), *ermF* (1.48%; p = 0.0004 and 1.28%; p = 0.0006) and *ermB3* (0.59%; p = 0.0002 and 1.76%; p = 0.003). *mphA* was also more abundant than *oleC* and *mphA* in 2- and 4-month-old infants. *ermF* amounts at 4 months of age were significantly lower compared to the *oleC* gene.

#### Other ARGs

*bacA* and *mcr1* were highly relevant in the initial pre-screening (Fig. 3G). Both showed a significant increase from 7 days to 2 and 4 months of age: *bacA* increased from 2.01 to 6.31% (p < 0.05) and then to 14.66% (p < 0.05), whereas *mcr1* increased from 0.74 to 1.53% (p < 0.05) and then decreased to 1.06% (p < 0.05).

#### Phenicol

To quantify phenicol-resistant genes, *catA1, cmlV* and *mdtL* were measured (Fig. 3H). The comparison between their abundance in each timepoint showed that at both 2 and 4 months of age, *catA1* (0.37% and 0.23%) was significantly lower than *mdtL* (3.96% and 2.75%; p = 0.003 and p = 0.002 for each time) and *cmlV* (3.06% and 4.44%; p = 0.001 and p = 0.006). The individual genes did not show any time-related fluctuations between sampling points (p > 0.05).

#### Quinolone

Quinolone-resistant genes *qepA* and *qnrB* showed a significant increase form 7 days (6.55% and 0.17%, respectively) to 2 months (23.44%; p = 0.01 and 0.26%; p = 0.05) and then again at 4 months of age (28.26%; p = 0.01 and 0.31%; p = 0.01). *qnrS1* was significantly lower in abundance than *qepA* and *qnrB* at all timepoints (p < 0.05) (Fig. 3I).

#### Tetracylcine

Antibiotic resistance to tetracycline was measured by the quantification of *tetA2, tetL2, tetO2,* and *tetW* genes (Fig. 3J). The *tetL2* gene increased significantly from 7 days (0.49%) to 2 months (1.77%; p = 0.01) and then again at 4 months (2.51%; p = 0.01). On the contrary, the *tetO2* gene decreased in abundance from 7 days (4.11%) to 4 months (1.95%; p = 0.05).

#### Vancomycin

The *vanA, vanB* and *vanHB* genes, conferring resistance to vancomycin, increased significantly over time (Fig. 3K). *vanA* increased from 0.73% at 7 days of age to 4.60% at 2 months (p = 0.001) and to 5.82% at 4 months (p = 0.0001). Similarly, *vanHB* increased significantly from 7 days (0.30%) to 2 months (2.13%; p = 0.001) and to 4 months of age (2.12%; p = 0.01). Finally, *vanB1* gene increased significantly from 7 days (0.33%) to 2 months (0.89%; p = 0.01), from 1 months (0.25%) to 2 months of age (p = 0.05), from 1 to 4 months (1.15%; p = 0.05) and from 7 days to 4 months (p = 0.01). At the end, the abundance of *vanA* is higher than *vanB1* (p = 0.039).

### Factors that modulate antibiotic resistance though time

#### Mode of delivery and antibiotic exposure at birth

**Fig. 4:**
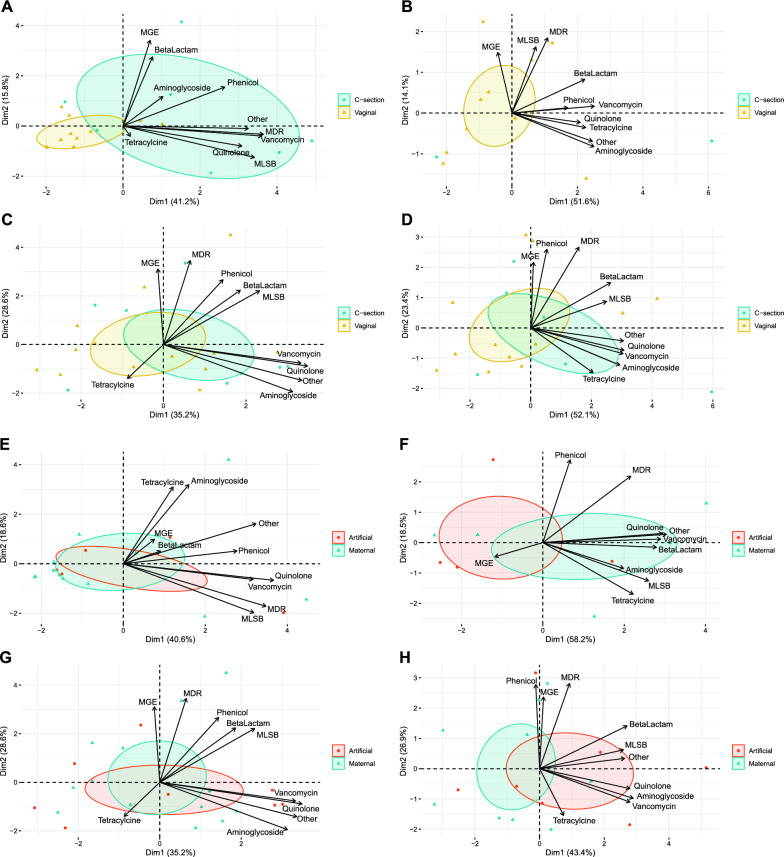
Association between antibiotic resistances and external factors. Principal Component Analysis (PCA) biplot depicting the relationship between the composition of antibiotic resistances and mode of delivery (**A**–**D**) and lactation (**E**–**H**) at: 7 days (**A** and **E**), 1 month (**B** and **F**), 2 months (**C** and **G**) and 4 months (**D** and **H**) of age. For delivery mode, blue ellipses represent the infants born by c-section and yellow ellipses the infants born vaginally. For lactation, red ellipses represent the infants who received artificial lactation and blue ellipses the exclusive breastfed infant. The black arrows are the group of ARGs that best explain the differences between the sample groups. Angles between the arrows represent correlations; acute angles represent positive correlations and obtuse angles represent negative correlations. Figure 3I represents the top 50 features with significant associations with external factors and time. Delivery_mode including C-section and Vaginal delivery. Lactation_mode includes exclusive breastfeeding and artificial lactation. Only significant associations (p < 0.05) bases on MaAsLin approach are shown

Infant’s mode of delivery exerted a strong influence on antibiotic resistance composition in 7-day-old infants, resulting in a significantly different antibiotic resistance composition for infants born vaginally or with c-section procedures (Fig. 4A–D). Hence, at 7 days of age most of the antibiotic resistances are associated with c-section birth (p < 0.001). The class of ARGs most contributive to the first PC component was MDRs, followed by vancomicyns, MLSBs, others, quinolones and phenicol. MGEs and genes conferring resistances to beta-lactamases, contributed mostly to the second PC component.

In 1-month-old infants, we observed that most of the ARGs, except for MGEs, were positively associated to c-section birth. Vancomycin-related genes and aminoglycoside-related ones were the main contributors to the ARG variation in our study population depending on mode of delivery (p < 0.001).

Similarly, at 2 months of age, infants born by c-section were also associated with the majority of the antibiotic resistances (p < 0.001) with the exception of tetracyclines, which were associated with vaginally birth. However, this pattern was reverted at 4-months of age, where again most antibiotic resistances were associated with c-section delivery, following the order of vancomycin, quinolone, others, aminoglycosides, beta-lactams, MLSB and tetracyclines (p < 0.001 for all). MDR-, phenicol- and MGE- related genes made a greater contribution to the second component (p < 0.001) and their association with c-section was slightly lower.

#### Infant feeding type

Infant feeding type (maternal breastfeeding or artificial lactation, which included the combination of breastfeeding and formula-feeding, and exclusive formula-feeding) was also found to influence the ARG profile in infants (Fig. 4E–H). At 7 days of age, vancomycin, MDR, other, MLSB, quinolone, and phenicol ARGs were highly associated with infants who received infant formula-feeding (p < 0.001). This pattern was maintained at 1 month of age, where beta-lactam- and tetracycline-resistant genes also gained importance in artificially fed infants. However, at this timepoint MGE was associated to breastfed infants. At 2 months of age genes classified as quinolones, vancomycins, aminoglycosides and others were strongly correlated to infants who received artificial lactation. Finally, 4-month-old infants also showed a significant contribution between artificial lactation (p < 0.001) and most ARGs, namely aminoglycoside-, vancomycin-, quinolone- and beta-lactam-resistant genes.

**Fig. 5:**
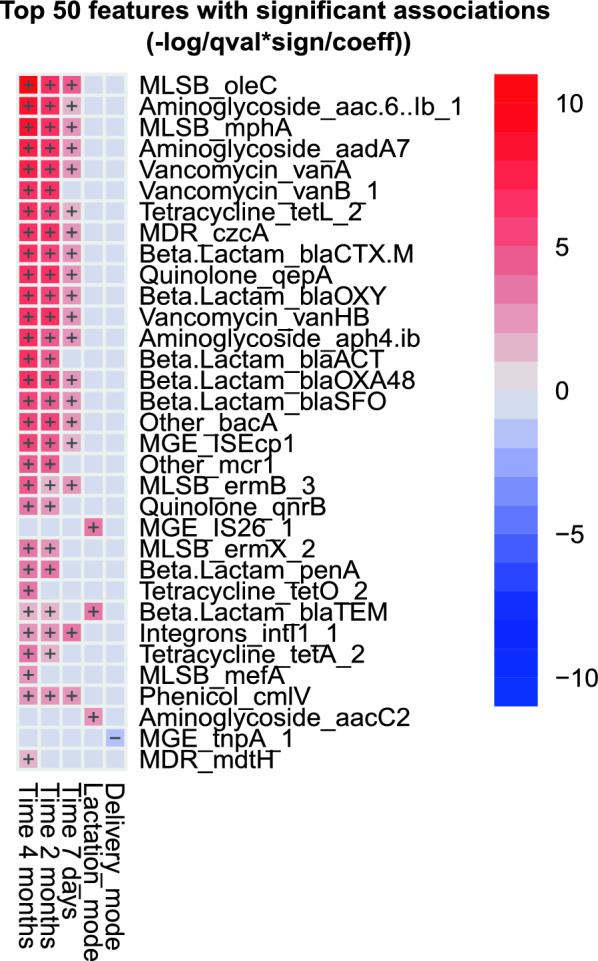
Top 50 features with significant associations with external factors and time. Delivery_mode includes c-section and vaginal delivery. Lactation_mode includes exclusive breastfeeding and artificial lactation. Only significant associations (p < 0.05) are shown

In addition, the MaAsLin approach allowed us to find specific associations (Fig. 5) when controlling for infant age and other variables (lactation and delivery). We observed an association of longitudinal samples with the establishment of resistance genes (very strong and rapid changes with respect to time). It is complex to have a marker associated since there are many changes associated with time and more in concert with the samples of 1, 2 and 4 months. Even though, we can find markers positively associated with delivery, such as MGE_tnpA_1, and also associated with lactation, such as aminoglycoside_aacC2, Beta-Lactam_blaTEM and MGE_IS26_1.

#### Environmental factors, microbiota and ARGs

Alpha and beta diversity analyses and taxa composition of microbiota are reported in Supplementary Fig. 2. Beta diversity analyses showed that microbial composition was significantly different between timepoints (7 days vs. 4 months: p = 0.006), and 1 month vs 4 months: p = 0.006). The GLMM explained the associations between the top 10 most abundant microbial genus, adjusted by environmental factors, on the antibiotic resistance gene load (Table 4). For the time categories, 1 and 4 months of age showed significant p-values (p = 0.001 and 0.003, respectively), indicating evidence that age has an effect on the ARGs composition. Variables related to breastfeeding and vaginal delivery also have significant p-values. From the 10 most abundant genera, *Bifidobacterium* (p = 0.042), *Streptococcus* (p = 0.002), and *Bacteroides* (p = 0.018) showed an influence on the ARG composition, meaning that the abundance of these species plays a crucial role on the composition of antibiotic resistances. The interaction between all environmental factors studied showed that breastfeeding and 4 months of age have a strong interaction and influence on the ARGs composition, followed by the same interaction with the addition of vaginal delivery, and the interaction of only that age and mode of delivery. Similarly, vaginal mode of delivery and 1 month of age also show a significant influence, higher than the interaction of that age and breastfeeding. These results highlight the importance of time on ARG composition among the other factors but elucidate that vaginal mode of delivery and breastfeeding can also drive the ARG composition, though likely in a time-related manner.

**Table 4:** Generalized lineal mixed model (GLMM) to explain the associations between the microbiota and environmental factors on the antibiotic resistance gene load

Variable	Wald value	p-value	Statistics
Time			
Time_4months	1.551	0.003	**
Time_1month	0.821	0.001	***
Microbiota (top 10 genus)			
*Bifidobacterium* spp.	2.528	0.042	*
*Streptococcus* spp.	3.060	0.002	**
*Bacteroide*s spp.	2.297	0.018	*
Environmental factors			
Lactation_Maternal	0.855	0.015	*
Delivery_Vaginal	0.582	0.005	**
Interactions			
Time_4months –- Lactation_Maternal	1.393	0.001	***
Time_4months –- Lactation_Maternal –- Delivery_Vaginal	0.438	0.009	**
Time_4months –- Delivery_Vaginal	0.366	0.007	**
Time_1month –- Delivery_Vaginal	0.332	0.001	***
Lactation_Materna –- Delivery_Vaginal	0.319	0.016	*
Time_1month –- Lactation_Maternal	0.221	0.018	*

Wald test was applied to assess the significance of the coeficients obtained with the GLMM. Only significant associations are shown. Signif. codes: (*): p < 0.05; (**): p > 0.001; (***): p > 0.0001. N = 19 of 7 days old infants, N = 11 of 1 month old infants and N = 18 of 4 months old infants

## Discussion

In this study, we investigated the dynamic evolution of antibiotic resistance genes in the early-life gut microbiome of infants, shedding light on ARG evolution as well as the factors influencing them. Our findings reveal a significant shift in the ARGs profile from 7 days to 4 months of age, indicating a complex trajectory of resistance development in the infant gut. This observation highlights the novelty of our research, as it underscores the importance of understanding the resistome dynamics in early life, a critical period for microbiome development.

One novel aspect of our study is the use of a high-throughput, quantitative approach to measure and quantify ARGs, providing a high-resolution view of ARG abundance. This approach offers advantages over other methods, such as amplicon sequencing or shotgun metagenomics, as it allows for precise quantification of specific resistance genes [45]. This precision is particularly valuable when monitoring changes in ARG abundance over time and in response to different factors. The same high-throughput qPCR approach employed in this study has until now, mostly been used for environmental resistome analyses, such as those of aquatic environments [46, 47], wastewater treatment plants [48], and soil and manure [49, 50], and, to a lesser extent, in farm and wild animal gut resistomes [51–53].

The examination of several genes in our study revealed novel information that enhances our understanding of the evolution of the infant resistome, shedding light on previously unexplored aspects of this important subject. We have observed that most antibiotic resistances increase through time, as 4-month category had a higher value, suggesting that the effect of age on the ARGs composition is more pronounced at that moment.

Comparing our findings with existing literature, we note several key insights. Firstly, our study highlights the emergence of mobile genetic elements (MGEs) as prominent contributors to ARGs in the early infant gut, particularly at 7 days of age. This finding aligns with previous research emphasizing the role of MGEs in disseminating resistance genes [11, 16, 54]. Our study found that MGE were higher in infants than in mothers in agreement with previous studies [16]. In addition, despite limited data on MGE, we also found higher MGE at 7 days compared to the later time points. This observation is also in agreement with a recent study where they found higher MGEs at 7 days compared with later time points using a metagenomic approach [55]. Research has indicated that during the initial stages of life, the presence of mobile genetic elements could be attributed to maternal factors or the specific family environment, which has the greatest impact by the time a child reaches 6 months of age [56]. In contrast, beta-lactam resistance genes exhibit complex patterns, with some genes increasing in abundance over time, while others experience shifts. These trends reflect the intricate dynamics of ARGs in response to selective pressures [15].

Abundance patterns of ARGs associated with quinolones, vancomycin, and aminoglycosides were significantly increased with 4-month-old samples. It has been described that the prescription of quinolones to mothers increases the risk of their offspring acquiring community-acquired, quinolone-resistant *E. coli* [57]. Vancomycin is prescribed during the perinatal period for treating colitis, *C. difficile*–induced diarrhea, and gram-positive bacterial infections [58]. Aminoglycosides, usually combined with a beta-lactam, are widely used for neonatal sepsis, and genes for acetylation, phosphorylation and adenylation conferring aminoglycoside resistance have been identified in infant gut microbiota [59]. However, the percentage of infants of our study that were exposed to antibiotics was very low, and none of them suffered from sepsis or colitis. All this consistently supports the notion that antibiotic exposure, either directly or indirectly, influences the infant resistome, and also that antibiotic resistances can occur even in the absence of antibiotic exposure [44].

Our study highlights the significant increase of tetracycline resistance over time. The resistance gene *tet* has been reported to be the most abundant and representative resistance genes in the infant gut resistome, possibly due to its prevalence in various bacteria species, such as Firmicutes and Bacteroidetes [60, 61]. It has also been reported that vertical transmission from the mother can be responsible for the acquisition of these resistance in infants [62–64]. Although tetracyclines are not used during pregnancy and early life, they are still extensively used in animals and, therefore, the environment and the diet may be sources of *tet* genes without direct exposure to the antibiotic [62, 65]. As a result, infants face a significant increase of tetracycline resistance over time.

Multidrug resistance in bacteria occurs by accumulation of ARGs on plasmids or transposons. Macrolide–lincosamide–streptogramin B (MLSB) is a significant multidrug-resistant phenotype usually related to staphylococci as it leads to methicillin-resistant *Staphylococcus aureus* (MRSA). Neonatal MRSA infections not only have high mortality and morbidity rates but also have long-term adverse effects on neonates [66]. The macrolide resistance gene *ermB* has previously been found to be prevalent in the infant gastrointestinal tract [67], correlating with our findings. Most *ermB* carriers turned out to be *Enterococcus* spp., and *Klebsiella* spp., from which some pathogenic species are considered multi-drug resistant bacteria. This study also reports that the *mcr1* gene, which confers resistance to colistin, increases significantly in the infant gut over time, and it has been previously identified in multidrug resistant *Salmonella enterica*, which causes acute diarrhea [68].

Our analysis also considers the influence of factors like mode of delivery and lactation on the resistome. The results presented in our study showed a significant difference in ARG composition in 7-day-old infants based on the mode of delivery. The alteration of the early gut microbiome due to delivery mode may contribute to differences in ARG composition, and some studies have elucidated the presence of ARGs in c-section born babies [9, 69, 70]. Moreover, the association of MDR genes with infants born via c-section is particularly noteworthy. A study by Yassour et al*.* [71] found that c-section delivery was associated with a higher risk of the infant gut microbiome being enriched with opportunistic pathogens carrying MDR genes [71]. This aligns with our findings, where MDR genes were prominent in c-section-born infants at 7 days of age. The differential association of tetracycline resistance genes in 2-month-old infants is of interest, as it suggests that factors beyond the mode of delivery may also contribute to ARG acquisition.

The influence of mode of lactation on ARG composition in infants, as demonstrated in our study, adds another layer to the complexity of early-life microbiota and resistance gene dynamics. Previous research has shown that breastfed infants exhibit a distinct gut microbiome and resistome profile [11, 72, 73] compared to formula-fed infants. Our findings are consistent with this observation, as we noted differences in ARG composition based on mode of lactation, particularly at 4 months of age. The prevalence of vancomycin, MDR, and other ARGs in artificially fed infants is concerning. These genes confer resistance to antibiotics that are crucial for treating various infections. The temporal changes observed in the association of specific ARGs with mode of lactation raise important questions about the mechanisms underlying these shifts.

The varying contributions of different ARGs at different time points emphasize the dynamic nature of early-life gut microbiota and its susceptibility to external influences. One-health approach methodologies are essential for deciphering the complex resistome structure in the microbiomes of humans, animals, and the environment. Further research is needed to explore the mechanisms behind these fluctuations and their potential long-term consequences.

Gut microbiota profiling was performed in a subset of our population to address the possible influence on the antibiotic resistance load. Interestingly, *Bifidobacterium* spp. showed the strongest effect on ARG composition, shedding light into the important role of these genus on the variation of antibiotic resistances in early life. In some studies, the negative correlation between *Bifidobacterium* abundance and ARG load has been described in infants from 7 days to 4 months of age [74]. Moreover, it has been found that *Bifidobacterium* genus themselves are less likely to possess ARGs than other taxa such as *Enterococcus, Streptococcus, Staphylococcus,* and *Bacteroides* genus [75, 76]. It is important to consider that as the infant grows, their microbiome diversifies, acquiring a more complex phylogenetic structure. This increased microbial diversity is accompanied by an increase in the diversity of the resistome. Although these findings are important, further metagenomic approaches should be performed in order to specifically assess the influence of the different bacterial taxa in the early infant gut on the abundance of resistances.

While this study has provided valuable insights into the relationship between environmental factors and the composition of ARGs, several limitations should be considered. The sample size in this study was limited to 72 samples from specific geographic locations, potentially restricting the generalizability of the findings. Moreover, our study population did not have a representative group of infants exposed to antibiotics during their early-life, and this was beneficial to study the more targeted effect of lactation; it would be interesting for next approaches to include this factor.

The high-throughput methodology employed to quantify ARGs was robust; however, it was confined to a specific set of ARGs. This may have overlooked certain aspects or variations in other ARGs and low abundance ARGs have been missed. Despite these limitations, this study serves as a foundational exploration into understanding the interplay between environmental factors and ARG composition. Future research could address these limitations by expanding sample diversity, employing multi-omics approaches for ARG characterization, and conducting longer longitudinal studies to unravel the temporal dynamics of ARGs in different environmental settings.

## Conclusion

Our investigation into the dynamics of the early-life gut resistome in infants has illuminated the intricate and evolving landscape of antibiotic resistance genes during this critical developmental phase. Our study provides valuable insights into the evolution of the infant gut resistome and the use of qPCR-based ARG quantification, alongside comprehensive analysis of factors like mode of delivery and lactation, contributes to our understanding of how resistance genes develop and change over time. The significant shifts in resistance gene composition over time and the differences according to infant feeding type emphasize the need for a nuanced understanding of early-life resistance dynamics. These findings have important implications for strategies aimed at mitigating antibiotic resistance in infants and underscore the need for continued research in this vital area of microbiome science.

### Supplementary Information

Supplementary material 1. Copy number of the ARGs quantified by HT-qPCR in the pre-screening.

Supplementary material 2. Copy number of the 48 ARGs, MGE and integrons quantified by HT-qPCR.

Supplementary material 3. Genus relative abundance of the 21 most abundant taxa in each sample.

Supplementary material 4. Figure 1. Statistical differences between ARGs. Wilcoxon statistic value (W) between between ARGs. (A) Aminoglycosides, (B) Beta-lactams, (C) Integrons, (D) MDR, (E) MGE, (F) MLSB, (G) Others, (H) Phenicol, (I) Quinolone, (J) Tetracycline and (K) Vancomycin. Only ARGs of the same antibiotc group with significantly different relative abundances are represented. p-value is indicated next to each dot.

Supplementary material 5. Figure 2. Microbiota analysis of the study population. A) Alpha diversity measures of observed, Chao1, ACE, Shannon and Simpson indexes. B) Beta-diversity analysis performed with a bray-distance based PCoA of the microbial composition, coloured by the age of infants. Significant differences were found between time of 7 days and time 1 month (p=0.003) and 4 months (P=0,009). C) Taxaplot representing the composition of the study population. Supplementary table 3 shows the genus relative abundance of the 21 most abundant taxa in each sample. N= 19 of 7 days old infants, N=11 of 1 month old infants and N=18 of 4 months old infants.

## Data Availability

The dataset supporting the conclusions of this article is available in the NCBI’s Sequence Read Archive (SRA) repository, BioProject ID PRJNA614975 (http://www.ncbi.nlm.nih.gov/bioproject/614975).

## Abbreviations

ARG: Antibiotic resistance genes
MGE: Mobile genetic elements
MDR: Multidrug resistance
MLSB: Macrolide–lincosamide–streptogramin B resistance
