# Supplementary figures and images for: Unravelling the evolutionary dynamics of antibiotic resistance genes in the infant gut microbiota during the first four months of life
Source: Ann Clin Microbiol Antimicrob. 2024 Aug 13;23:72. doi: 10.1186/s12941-024-00725-z (PMC11323388; doi:10.1186/s12941-024-00725-z)

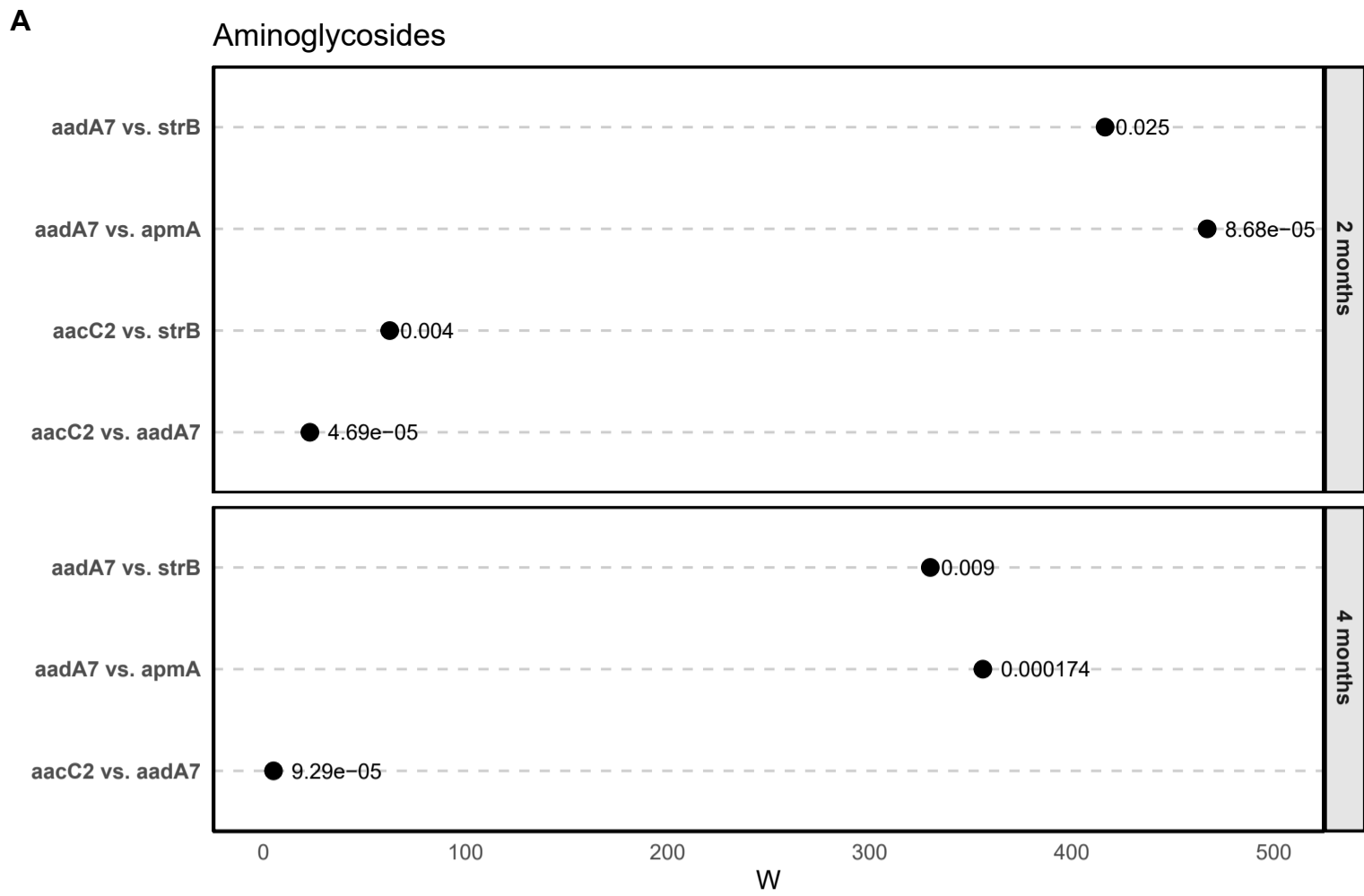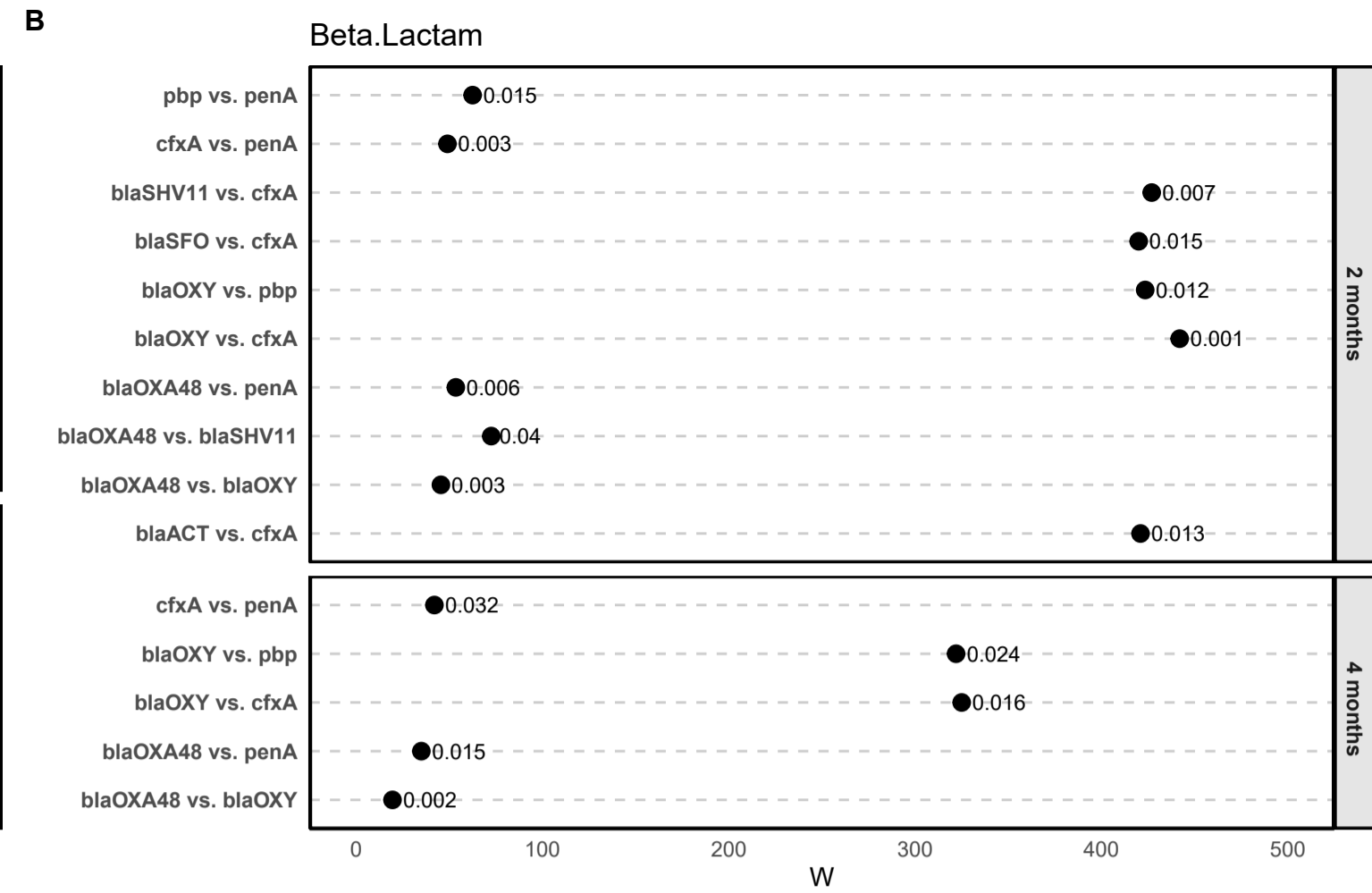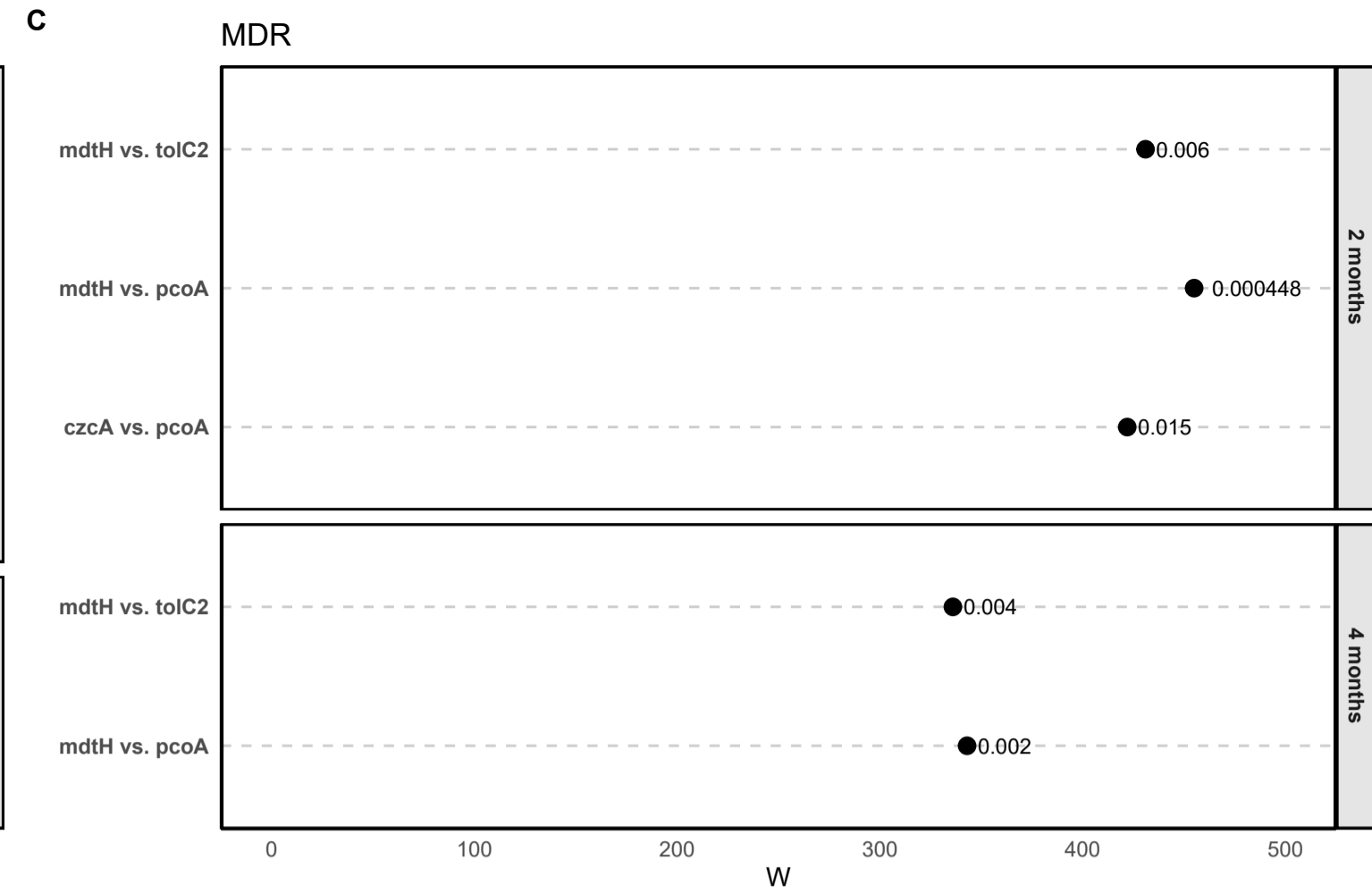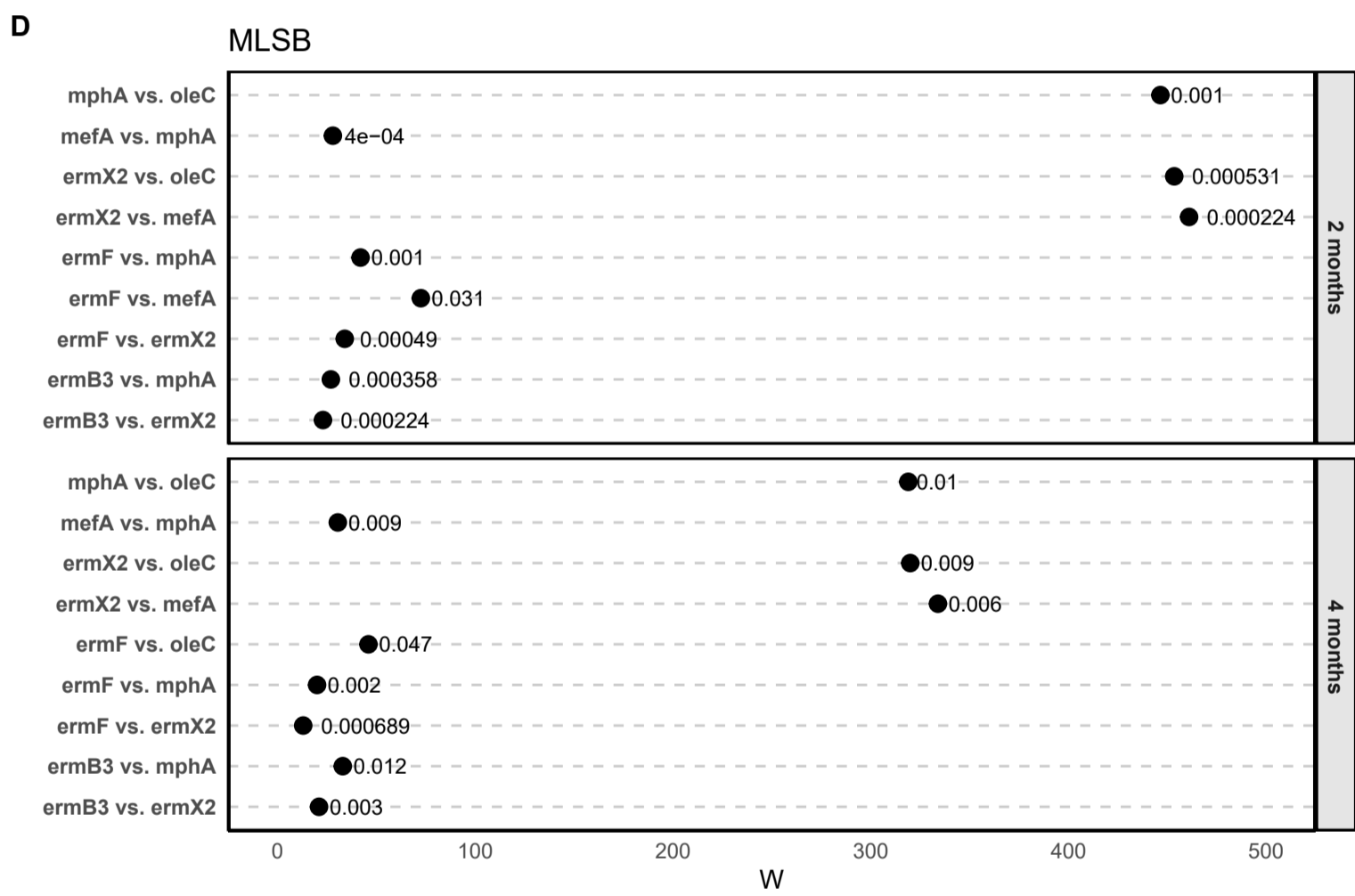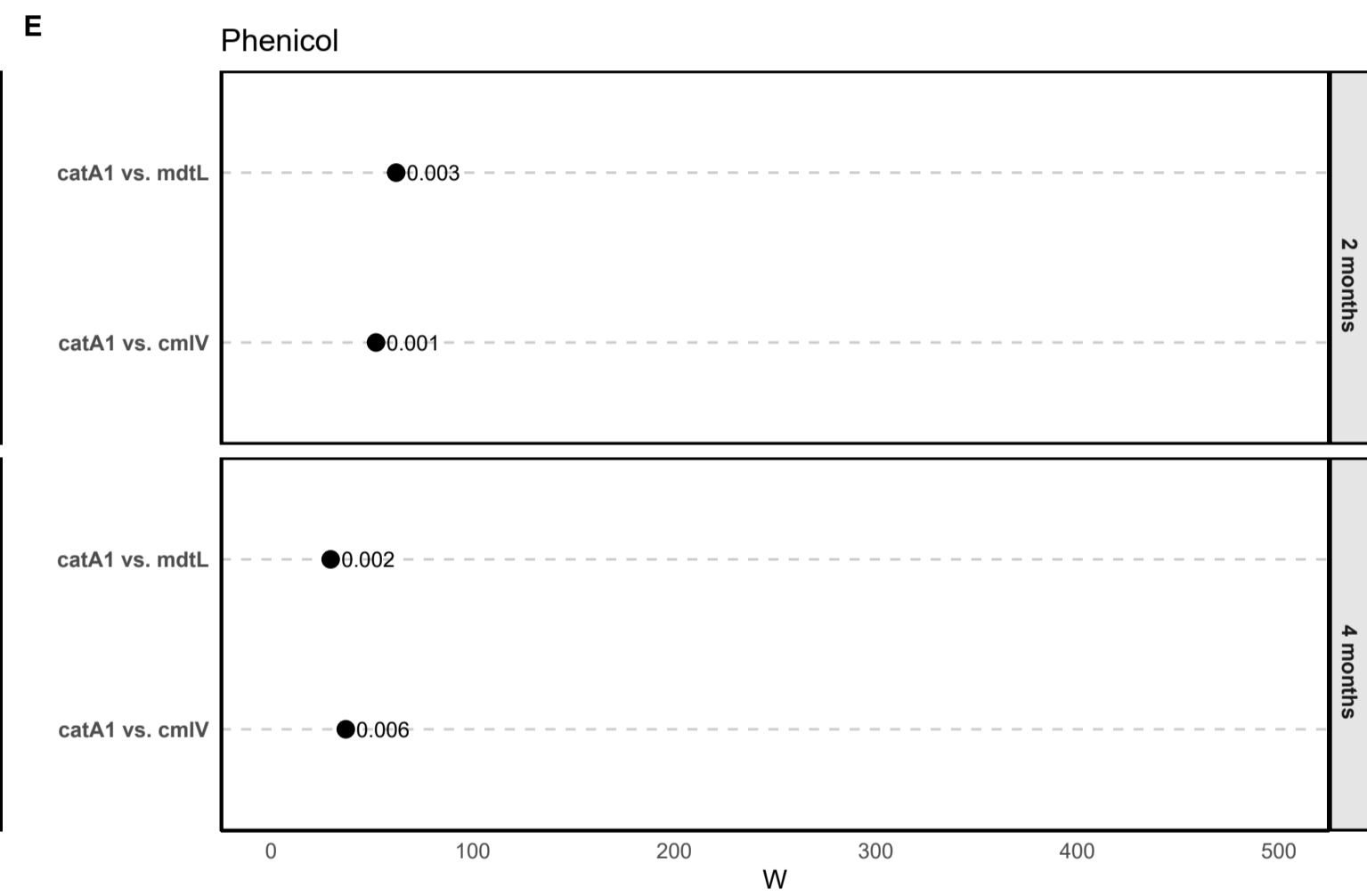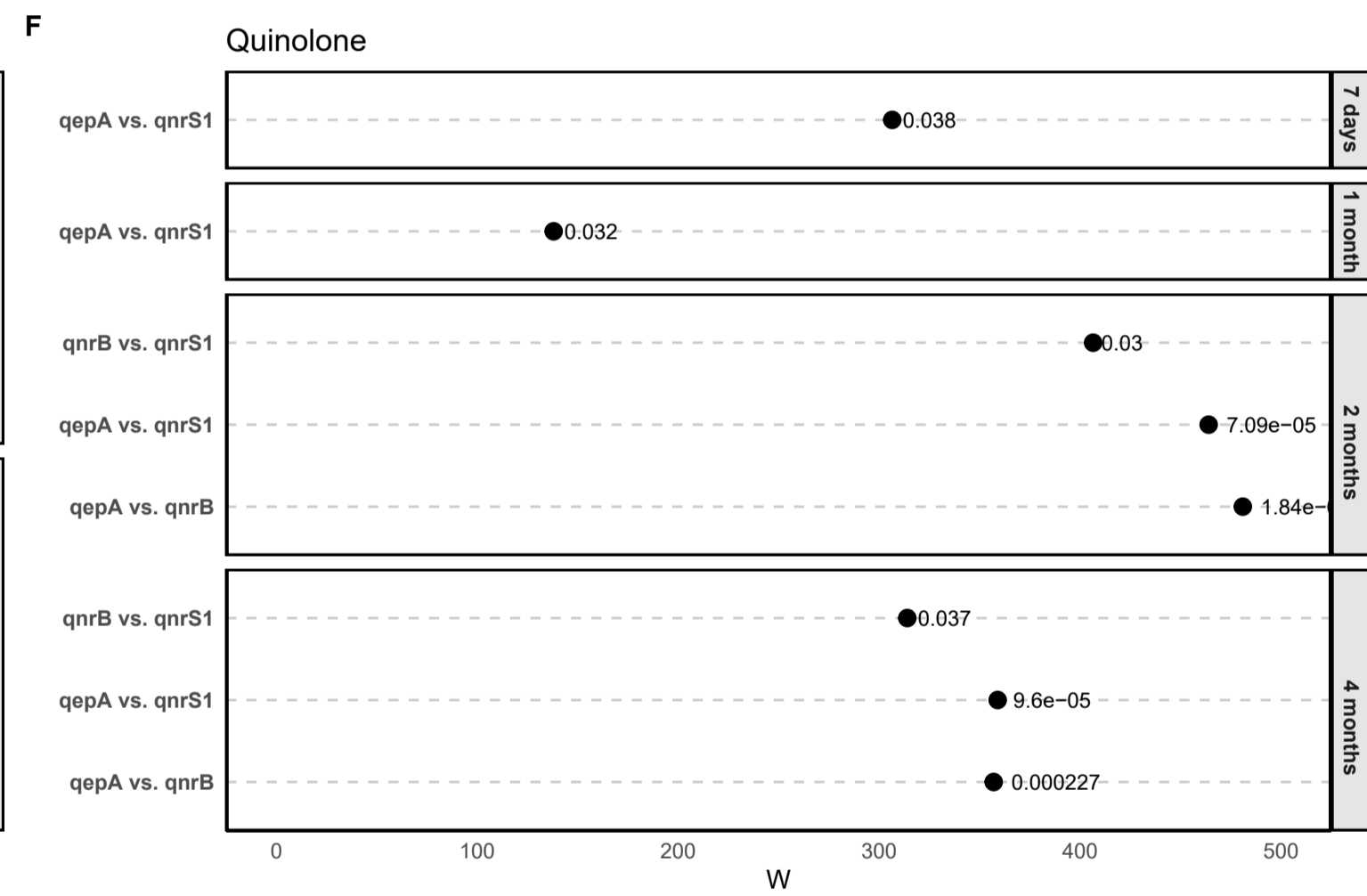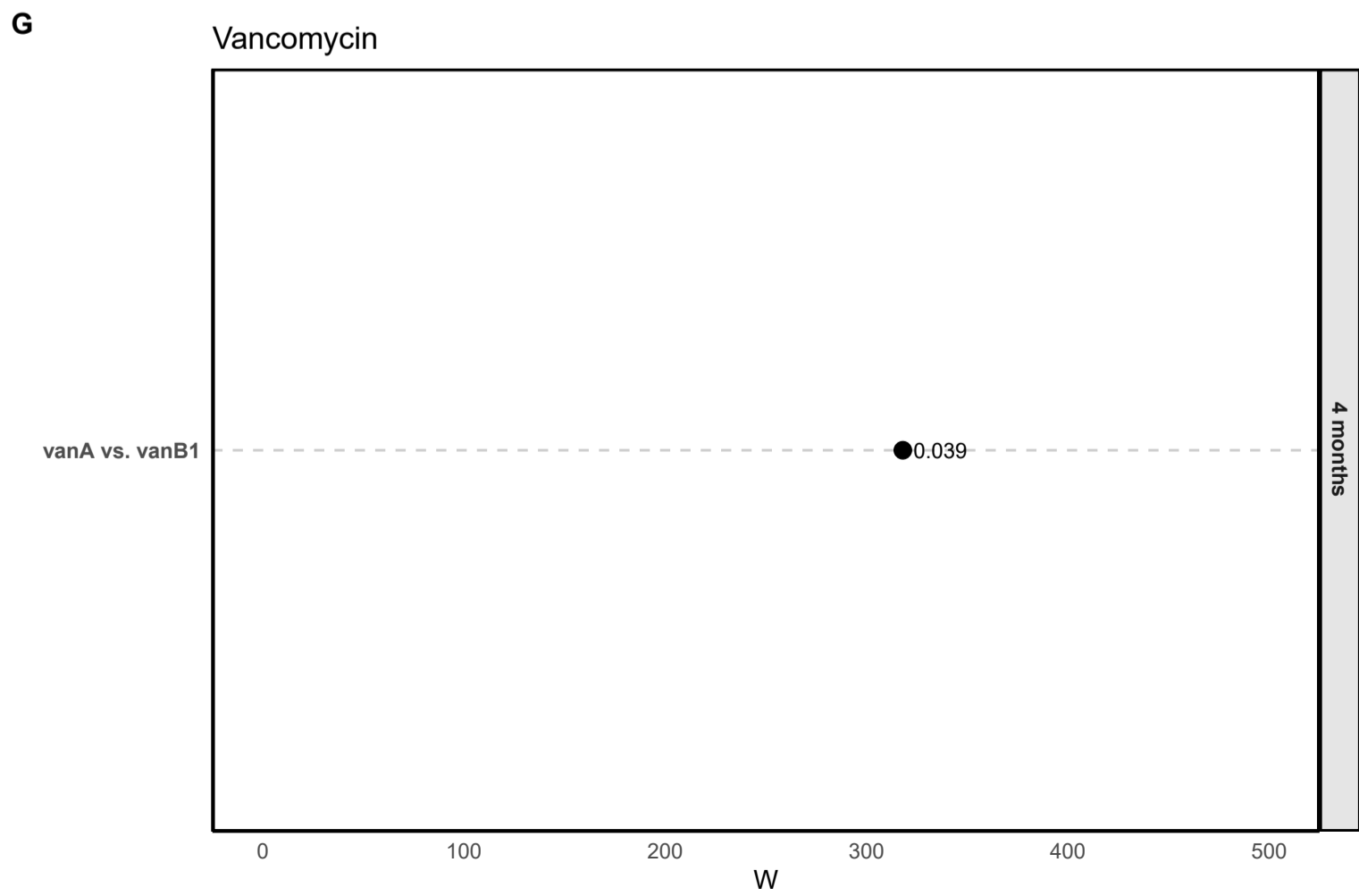

Supplement: Supplementary file 4 — Supplementary material 4. Figure 1. Statistical differences between ARGs. Wilcoxon statistic value (W) between between ARGs. (A) Aminoglycosides, (B) Beta-lactams, (C) Integrons, (D) MDR, (E) MGE, (F) MLSB, (G) Others, (H) Phenicol, (I) Quinolone, (J) Tetracycline and (K) Vancomycin. Only ARGs of the same antibiotc group with significantly different relative abundances are represented. p-value is indicated next to each dot. [file 12941_2024_725_MOESM4_ESM.pdf]

**A**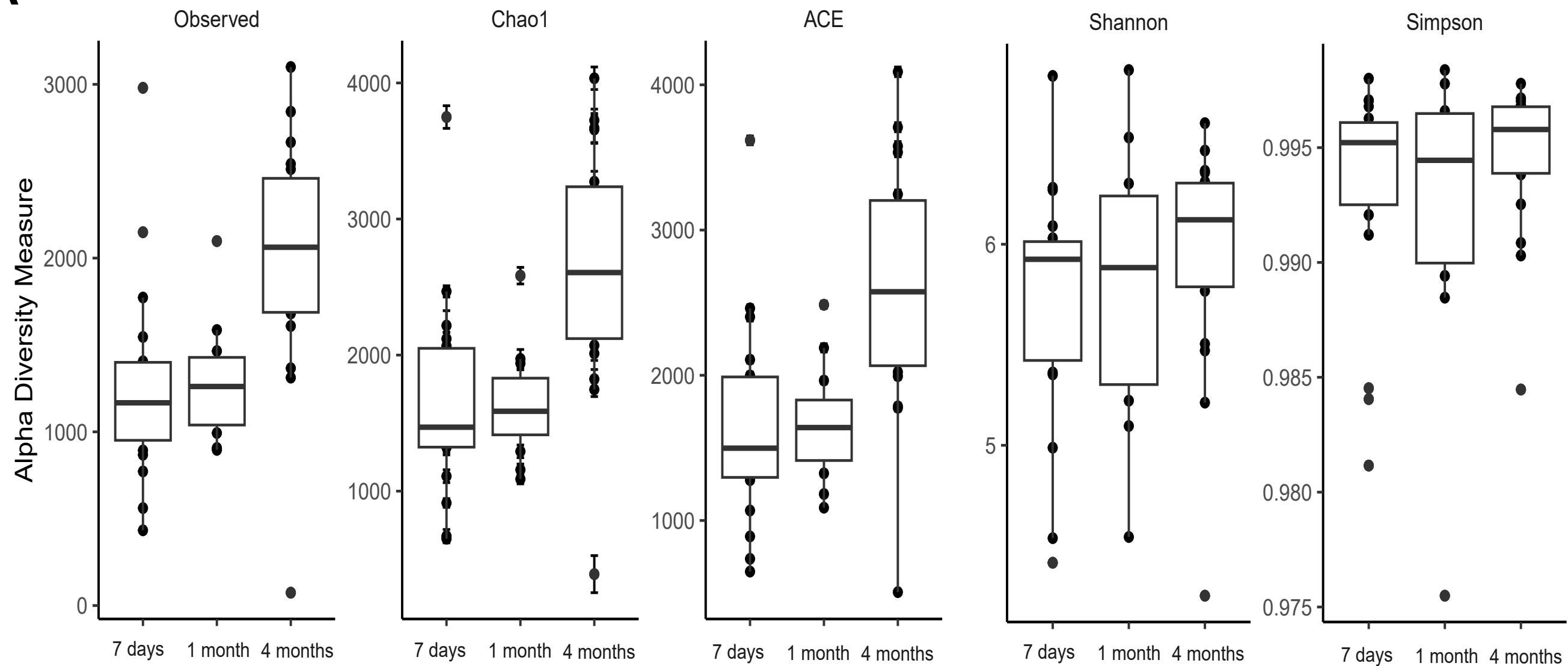**B**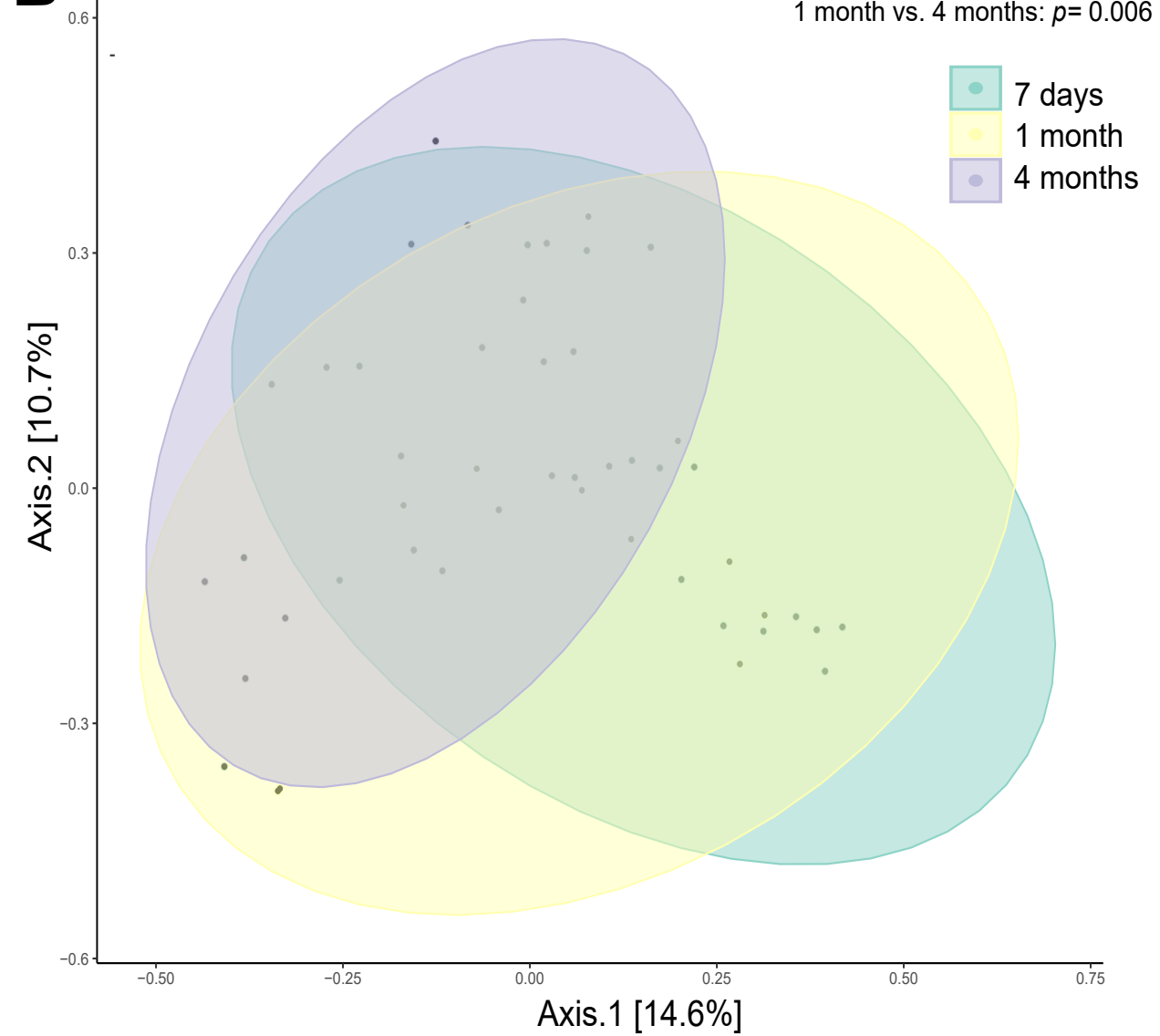**C**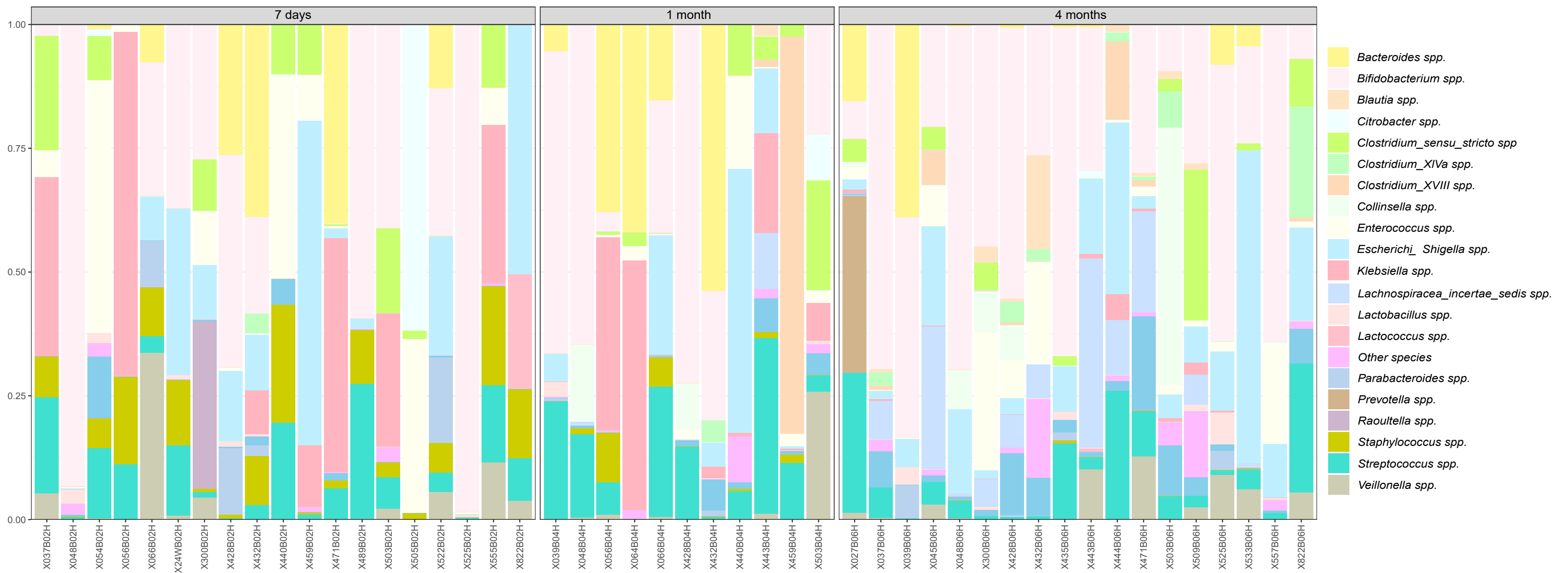

Supplement: Supplementary file 5 — Supplementary material 5. Figure 2. Microbiota analysis of the study population. A) Alpha diversity measures of observed, Chao1, ACE, Shannon and Simpson indexes. B) Beta-diversity analysis performed with a bray-distance based PCoA of the microbial composition, coloured by the age of infants. Significant differences were found between time of 7 days and time 1 month (p=0.003) and 4 months (P=0,009). C) Taxaplot representing the composition of the study population. Supplementary table 3 shows the genus relative abundance of the 21 most abundant taxa in each sample. N= 19 of 7 days old infants, N=11 of 1 month old infants and N=18 of 4 months old infants. [file 12941_2024_725_MOESM5_ESM.pdf]
